# Multi-scale-average-filter-assisted level set segmentation model with local region restoration achievements

**DOI:** 10.1038/s41598-022-19893-z

**Published:** 2022-09-24

**Authors:** Lutful Mabood, Noor Badshah, Haider Ali, Muhammad Zakarya, Aftab Ahmed, Ayaz Ali Khan, Lavdie Rada, Muhammad Haleem

**Affiliations:** 1 Department of Mathematics, University of Peshawar, Peshawar, Pakistan; 2 Department of Basic Sciences, University of Engineering and Technology, Peshawar, Pakistan; 3 Department of Computer Science, Abdul Wali Khan University, Mardan, Pakistan; 4 Department of Computer Science, University of Lakki Marwat, Khyber Pakhtunkhwa, Pakistan; 5 Biomedical Engineering Department, Bahcesehir University, Besiktas, Istanbul, Turkey; 6 Department of Computer Science, Kardan University, Kabul, Afghanistan

**Keywords:** Computer science, Software

## Abstract

Segmentation of noisy images having light in the background it is a challenging task for the existing segmentation approaches and methods. In this paper, we suggest a novel variational method for joint restoration and segmentation of noisy images which are having intensity and inhomogeneity in the existence of high contrast light in the background. The proposed model combines statistical local region information of circular regions centered at each pixel with a multi-phase segmentation technique enabling inhomogeneous image restoration. The proposed model is written in the fuzzy set framework and resolved through alternating direction minimization approach of multipliers. Through experiments, we have tested the performance of the suggested approach on diverse types of synthetic and real images in the existence of intensity and in-homogeneity; and evaluate the precision, as well as, the robustness of the suggested model. Furthermore, the outcomes are, then, compared with other state-of-the-art models including two-phase and multi-phase approaches and show that our method has superiority for images in the existence of noise and inhomogeneity. Our empirical evaluation and experiments, using real images, evaluate and assess the efficiency of the suggested model against several other closest rivals. We observed that the suggested model can precisely segment all the images having brightness, diffuse edges, high contrast light in the background, and inhomogeneity.

## Introduction

In the domain of image processing, the two terms image segmentation and image restoration are closely related tasks with various applications in engineering, technological fields, pathology, astronomy, advanced driver assistance systems, etc. Utilizing aspects of image restoration to efficiently segment objects with, relatively, a high degree of noise, blur, missing pixels, or inhomogeneity is a commonly encountered task. Restoration and segmentation task can be simultaneously combined into a scheme or jointly represented into a minimization functional. Due to the specific properties of the images and segmentation requirements, the combined joint schemes result to be more useful and accurate.

The restoration task was the first task encountered after photography development. The first restoration approach was towards noise removal followed by deconvolution, inpainting etc. One of the most successful models for additive noise removal was total variational (TV) based model introduced by Rudin Osher Fatemi (ROF)^1^ in 1992. The task of image denoising restoration is the evaluation of a desired original image $u({\textbf {x}}):\Omega \longrightarrow \mathbb {R}$ given $u_0({\textbf {x}})=u({\textbf {x}})+\eta ({\textbf {x}})$ with $u_0({\textbf {x}}):\Omega \longrightarrow \mathbb {R}$ corrupted image with additive noise $\eta ({\textbf {x}})$. To reconstruct $u({\textbf {x}})$ from the observed degraded image $u_0({\textbf {x}})$, the ROF method utilizes the TV norm as a data regularization term. The energy functional of the ROF model^1^ is illustrated mathematically in the following Eq. (1):

1 $$\begin{aligned} F^{ROF}(u({\textbf {x}}))=\lambda \int _{\Omega }(u_0({\textbf {x}})-u({\textbf {x}}))^2d{\textbf {x}}+\int _{\Omega }|\nabla u({\textbf {x}})|d{\textbf {x}}, \end{aligned}$$

where $\lambda$ is a regularization parameter, $\Omega$ is the image domain and $\int _{\Omega }|\nabla u({\textbf {x}})|d{\textbf {x}}$ characterizes the total variation of $u({\textbf {x}})$. The minimization of the Eq. (1) leads to a PDEs grounded methods and approach which has been additionally drawn-out and revised to the separation of multiplicative noise through implementing the logarithm transformation or other restoration techniques. In fact, one of the key and foremost advantages of the ROF model^1^ is that it conserve and store the edge information while smoothing the leftover regions^2^. This quality is of help in case the image will further be segmented. In order to remove the staircase effect or better performance different methods have been introduced^3–8^. Most of these techniques use high order derivative terms to obtain more details and reduce the staircase effect. The data fidelity term $\int _{\Omega }(u_0({\textbf {x}})-u({\textbf {x}}))^2d{\textbf {x}}$ used for ROF model shows effectiveness in case of Gaussian additive noise. Note that, for removing other types of noises, such as Poisson noise, impulse noise, etc., the fidelity term must change accordingly^9,10^. The denoising problem can be extended to image restoration by introducing a blurry linear operator $\varphi$ such that $u_0({\textbf {x}})=\varphi ({\textbf {x}}) u({\textbf {x}})+\nu ({\textbf {x}})$. As the restoration problem has two unknown, $\varphi$ and *u*, dealing with it is harder. For image restoration problem, local spatial convolution filters of both one-dimensional and two-dimensional signals filters in combination with denoising techniques has been suggested and designed based on the quantitative properties of the image.

On the other hand, image segmentation is, in fact, one of the basic and crucial tasks in the arena of image processing. This should be noted that image segmentation mainly focuses to split the presented image into various meaningful regions and, subsequently, obtain some useful information^11–13^, such as the distinction between foreground and background or object/feature separation. In general, image segmentation is largely implemented as a pre-processing and/or post-processing technique combined or coupled with other techniques such as image restoration, image/pattern recognition, etc. In this paper the word “inhomogeneous” refers to such images in which objects pixels values changes slightly from one side to another means having no fix value for an entire object or objects having diffuse edges which make segmentation problem difficult. The “Global information” means the average of all pixels values of an image/object. While “statistical local region information” refer when we take K $\times$ K window size matrix from an image and dealt with it by using statistical terms like mean, variance etc. In considering local information we take small region information which are usually taken in the form of circle using simple circle equations that is referred as circular regions. Moreover, we implement and make use of the level set approach which is assumed as a powerful numerical technique for image segmentation and analysis. In order to tackle with all the aforementioned problems we introduce a new model which consists of:

- coupling into an energy functional the desired restore image and the filtered image into a single energy functional for segmentation purposes using a fuzzy membership function;
- extending the Cai model^14^ to a new variation segmentation approach that has the capability to restore inhomogenous images by adapting image global information to statistical local region information of circular regions centered at each pixel with a multi-phase segmentation technique;
- introducing a variational image segmentation approach and image restoration approach which are capable to handle the segmentation of images with high contrast light in the background;
- we propose a new model which takes the advantages from both restoration, as well as, image filtering for segmentation purposes using the fuzzy membership function in difference with Cai model^14^, which utilizes the restored image obtained from ROF formulation; and
- through wide empirical analysis on different classical datasets of images, we ascertained that the suggested model is precise and effective, particularly, in images that have brightness, diffuse edges, high contrast light in the background, and inhomogeneity.

The remainder discussion if this paper is organized as deliberated in next sentences. In ”Related work” section, we offer a summary and overview of state-of-the-art image segmentation techniques. In “Machine learning for image segmentation” section, we deliberate various machine learning techniques and their role in the image segmentation. In “[Sec Sec4]” section, we detail the suggested revised and new segmentation model and alter it to vector-valued images. In “Experimental results” section, we assess and evaluate the performance of the suggested approach with other state-of-the-art segmentation approaches using different real-world and synthetic images datasets. In the assessment, we consider various images having brightness, diffuse edges, high contrast light in the background, and inhomogeneity. Conclusions are drawn in “Conclusions and future work” section along with several guidelines for future investigation and research.

## Related work

One of the most ambitious and stimulating tasks in image segmentation is intensity inhomogeneity. Problems, for instance, artificial illumination and non-uniform daylight can cause imperfection of acquisition which leads to image inhomogeneity. Intensity inhomogeneity highly affects the image segmentation precision due to the overlap of background and foreground. In the last decade, many promising algorithms and methods were introduced to tackle this problem^15–20^. However, all those methods have limitations and are unable to tackle severe intensity inhomogeneity and work for images with specific properties^21,22^. For a better understanding of their limitation, we will shortly revise and comment on some state-of-the-art approaches and techniques. In summation to the intensity-based approach, we will discuss the widely used deep learning-based methods and well-known techniques for tackling the low-level computer vision problems, for instance de-noising and artifact removal.

The main image segmentation techniques can be classified as: (i) edge-based segmentation approaches; and (ii) region-based segmentation techniques. The edge-based models^13,23,24^ incorporate edge detector functions which channelize the movement of active contour in the directions of the boundaries. Such functions rely on the gradient of the image data. The region-based models^11,16,17^ utilize region information, such as variance, mean etc., to move the contour in the directions of the object’s boundary. The edge-based models utilize the image local information to be unable good performance in noisy images and ignore the objects having diluted boundaries. On the other hand, the region-based methods and models are unable to tackle the intensity inhomogeneity. This is due to the fact that the intensity inhomogeneity is the local property of images rather than a global one. One of the benefits of region-based techniques is that these approaches are potentially less sensitive to the noise and outliers due to which their segmentation results are better in noisy images. In fact, the majority of the region grounded models are approximations to the milestone Mumford–Shah (MS) energy functional^12^. Among all of them, active contour without edges suggested by the Chan and Vese (CV)^11,12^ gained much popularity in the literature due to its simple implementation. In terms of the CV model, the energy functional is given and illustrated in Eq. (2):

2 $$\begin{aligned} F^{CV}(c_1,c_2,\Gamma )= & {} \mu (Length(\Gamma ))\nonumber \\&+\lambda _1\int _{inside(\Gamma )}|u_0({\textbf {x}})-c_1|^2d{\textbf {x}}\nonumber \\&+\lambda _2\int _{outside(\Gamma )}|u_0({\textbf {x}})-c_2|^2d{\textbf {x}}, \end{aligned}$$

where $u_0({\textbf {x}})$ is the given image, $\Gamma$ denotes smooth and segmented curve, $\mu$, $\lambda _1$ and $\lambda _2$ are positive parameters (to be tuned accordingly), $c_1$ and $c_2$ are the mean intensities of $u_0({\textbf {x}})$ inside and outside of the $\Gamma$, correspondingly. Although, the CV model is commonly used and it has promising results for additive Gaussian noise, however, its limitation can be easily observed in cases the image suffers from intensity inhomogeneity^25,26^. This drawback is due to the utilization of the global information of images and ignoring local features information^27^. To enhance the CV model for inhomogeneity image segmentation the Local Binary Fitting (LBF) model^16^ was introduced. The LBF model hires a kernel function to locate the local intensity information of images and embeds this information and statistics into a region-based active contour model and level set formulation^28,29^. In terms of the LBF model, the energy functional is given as illustrated in Eq. (3):

3 $$\begin{aligned} F^{LBF}(\Gamma , g_1, g_2)= & {} \lambda _1\int _{\Omega }\int _{inside(\Gamma )}K_{\sigma }({\textbf {x}}-{\textbf {y}})|u_0({\textbf {y}})-g_1({\textbf {x}})|^2d{\textbf {y}}d{\textbf {x}}\nonumber \\&+\lambda _2\int _{\Omega }\int _{outside(\Gamma )}K_{\sigma }({\textbf {x}}-{\textbf {y}})|u_0({\textbf {y}})-g_2({\textbf {x}})|^2d{\textbf {y}}d{\textbf {x}}, \end{aligned}$$

whereas the variables $\lambda _1$, $\lambda _2$ are constants, $K_{\sigma }$ represents the Gaussian kernel with standard deviation ($\sigma$). Furthermore, the variables $g_1$ and $g_2$ characterizes the two smooth functions that, in fact, approximate the local details and statistics of the image inside and/or outside of the $\Gamma$, correspondingly. Although, the LBF model can cope with the intensity inhomogeneous; nevertheless, this model is very sensitive to the initial contours. Moreover, changes on initial contour can potentially lead the LBF model to produce undesirable segmentation results. Therefore, to further improve the segmentation of intensity inhomogeneity images and for bias field correction, Li et al.^30^ suggested a new region based variational model^31,32^. The authors in Ref.^30^ defined an objective function for K-means clustering, which is weighted, in a locality close to every point, with the centers of the clusters and having a multiplicative component that, in fact, computes and estimates the bias within the locality. Subsequently, then the proposed function is amalgamated over the whole environment and embedded into a level set formulation. Even though, the method suggested by Li et al.^30^, overcomes the existing ones, still the method can not deal with high image inhomogeneity, as similar to the other methods, the method is grounded on the laying claim that every intensity inhomogeneous image is, in fact, homogeneous within a small region. Another problem with those methods is that there is no prediction on the scale of the homogeneous region. Dealing with serious and hard intensity inhomogeneity and tuning the scale, in particular, for inhomogenous regions may potentially cause undesired results. Taking into account these problems, Wang et al.^20^ suggested a multi-scale local (MSL), and region oriented, system and model for segmentation of intensity inhomogeneous images. With the assumption that the desired neat image $u({\textbf {x}})$ is vitiated and damaged by the additive noise $\eta ({\textbf {x}})$ and the intensity inhomogeneity $\varphi ({\textbf {x}})$, then the obtained image $u_0({\textbf {x}})$ is described as given by Eq. (4):

4 $$\begin{aligned} u_0({\textbf {x}})= \varphi ({\textbf {x}}) u ({\textbf {x}})+\eta ({\textbf {x}}). \end{aligned}$$

A generally accepted assumption is that the intensity inhomogeneity is a tardily changing and varying component over the entire image and it is constant within a small local region. The target is to acquire the corresponding clean image $u({\textbf {x}})$ which is impressed and disturbed by both the noise and intensity inhomogeneity. To achieve this, the MSL model defines a local region in a circular shape for capturing local information and statistics and then some kind of mathematical and statistical assessment is done on those local circular arenas for each and every pixel utilizing multi-scale low-pass filtering. Assuming $\hat{u}({\textbf {x}})=\varphi ({\textbf {x}}) u({\textbf {x}})$, we have the following relationship:

5 $$\begin{aligned} u_0({\textbf {x}})=\hat{u}({\textbf {x}})+\eta ({\textbf {x}}). \end{aligned}$$

Applying Eq. (1) it is easy to recover $\hat{u}({\textbf {x}})$ and then consider it as a given image which is only suffered from intensity inhomogeneity and free from noise. Thus, the problem reduces into finding $u({\textbf {x}})$ from $\hat{u}({\textbf {x}})=\varphi ({\textbf {x}}) u({\textbf {x}})$ where $\varphi ({\textbf {x}})$ is the intensity inhomogeneity. After applying the logarithmic transformation, we obtained:

6 $$\begin{aligned} \log (\hat{u}({\textbf {x}}))=\log (\varphi ({\textbf {x}}))+\log (u({\textbf {x}})). \end{aligned}$$

As both the inhomogeneity layer $\varphi ({\textbf {x}})$ and the clean image $u({\textbf {x}})$ are unknown directly finding the clean image $u({\textbf {x}})$ from Eq. (6) it is impossible. To defeat this difficulty. Wang et al.^20^ suggested a multi-scale average filter. The local circular regions are defined in order to make the model more adaptable in capturing intensity information in the local region of a given pixel. To examine and investigate the information of the local circular region at each center pixel $\mathbf {x}$ of the, particular, given image $\hat{u}$ the multi-scale average filter is designed in the following form as illustrated in Eq. (7):

7 $$\begin{aligned} MSF_{i}(\mathbf {x})={1\over n}\sum _{\mathbf {y}\in F_{\mathbf {x},i}}\hat{u}(\mathbf {y}), \end{aligned}$$

where, the subscript *i* is the radius of the local circular region and this can also be characterized as a scale parameter. Furthermore, the variable *n* symbolizes the total amount of pixels within that particular local circular region $F_{\mathbf {x},i}$ with center $\mathbf {x}$; and is subsequently defined by the following Eq. (8):

8 $$\begin{aligned} F_{\mathbf {x},i}=\{\mathbf {y}: \sqrt{(\mathbf {y}_1-\mathbf {x}_1)^2+(\mathbf {y}_2-\mathbf {x}_2)^2} \le i\}. \end{aligned}$$

Furthermore, $M_k(\mathbf {x})$ is taken to be the mean of the multi-scale average filter and it is characterized as illustrated using Eq. (9):

9 $$\begin{aligned} M_k(\mathbf {x})=\frac{1}{k}\sum _{i=1}^k MSF_{i}(\mathbf {x}), \end{aligned}$$

where *k* represents the entire amount of the scales and this needs to be tuned properly according to the images. It may be noted that in case the value of the variable *k* is little then very elite circular regions will be investigated for every center pixel which may lead to an unfavorable result. Similarly, on the other hand if in case the value of the variable *k* is taken very ample then it will potentially increase the computational cost due to the fact that too many local circular regions will consider for every center pixel value. By replacing $\varphi (\mathbf {x})$ in Eq. (6) by $M_k(\mathbf {x})$, we get the following relationship:

10 $$\begin{aligned} \log (\bar{u}(\mathbf {x}))=\log (\hat{u}(\mathbf {x}))-\log (M_k(\mathbf {x}))+\log (M_N). \end{aligned}$$

In fact, this should be noted that $\bar{u}$ is an approximation to the clean image or intensity inhomogeneity free image *u*, whereas $M_N$ is a constant, in fact normalized, to conserve the mean intensity of $\bar{u}$. Furthermore, Eq. (10) can be represented in an equivalent form to decrease the computational cost as:

11 $$\begin{aligned} \bar{u}(\mathbf {x})=\hat{u}(\mathbf {x})M_N/M_k(\mathbf {x}). \end{aligned}$$

**Figure 1:**
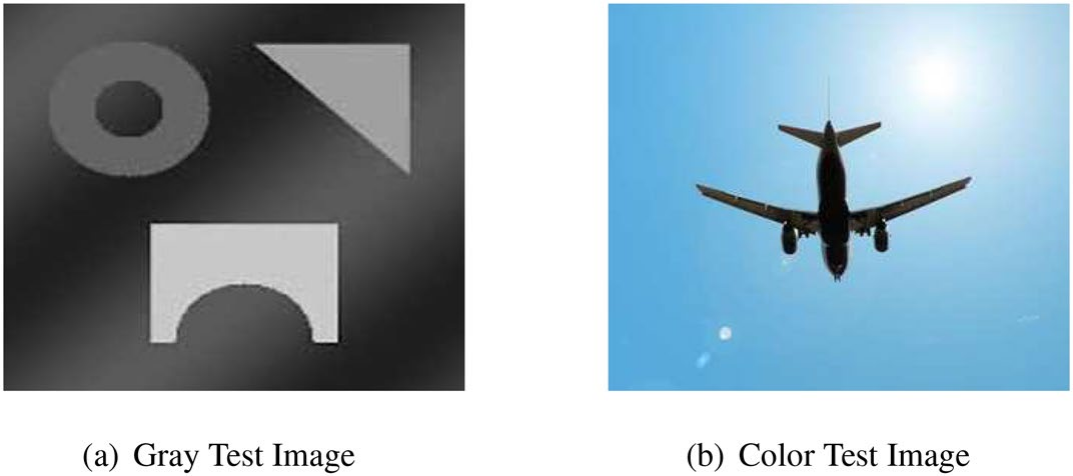
Two test images: the first one is gray-scale which is suffered from intensity inhomogeneity while the second one is color image having brightness in the background. These inhomogeneous images are taken from Cai et al.^14^ and are publicly available at the kaggle website^33^.

**Figure 2:**
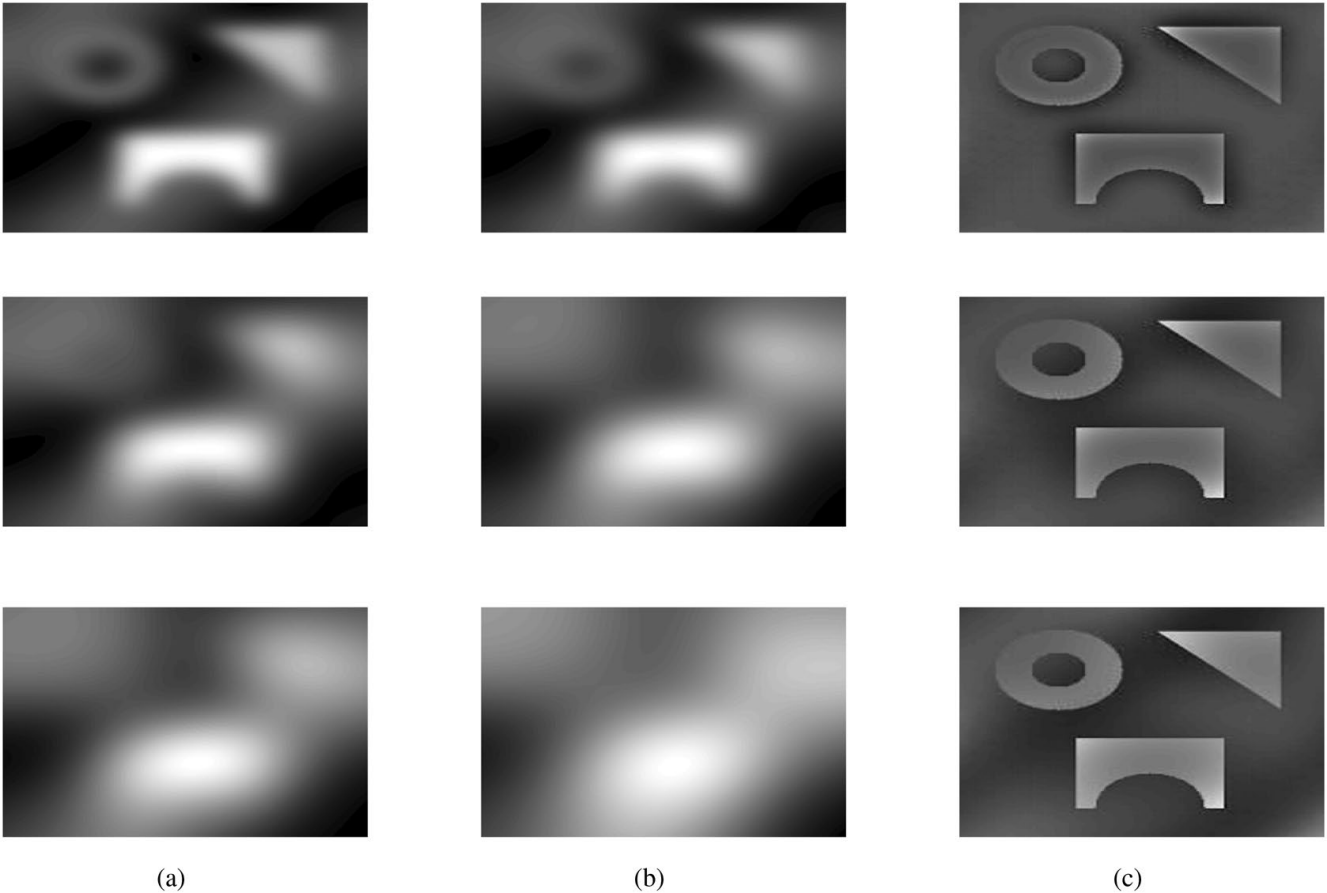
A demonstration of the dual filter formulation on gray image suffered from intensity inhomogeneity with different values of *k*. The first column (**a**) shows the inhomogeneity covered by first filter, the second column (**b**) shows the inhomogeneity covered by the second filter, and the last column (**c**) shows the inhomogeneity-free images. Furthermore, the first row deliberates the outcomes for $k=10$, the second row deliberates the outcomes for $k=20$, and the last row demonstrates the results for $k=30$.

**Figure 3:**
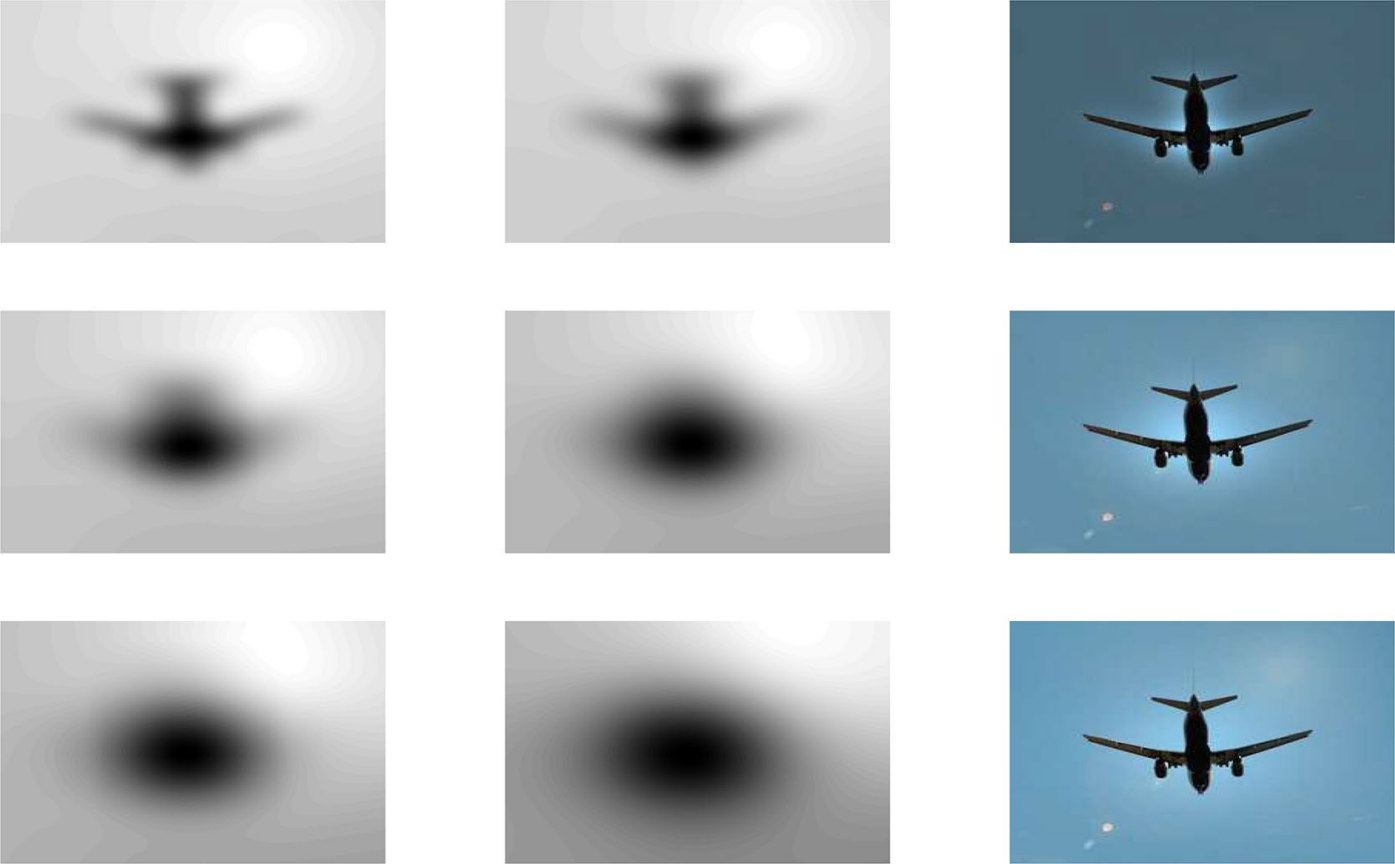
A demonstration of the dual filter formulation on color image of plane having high brightness in background, with different values of *k*. The first column shows the inhomogeneity covered by first filter, the second column shows the inhomogeneity covered by the second filter, and the last column demonstrates the inhomogeneity-free images. The first row deliberates the outcomes for $k=10$, the second row deliberates the outcomes for $k=20$, and the last row demonstrates the obtained results for $k=30$. These inhomogeneous images datasets are collected from the Goldstein et al.^6^, Cai et al.^14^ and are publicly available at the kaggle repository^33^.

In fact, Eq. (11) represents an approximation of inhomogeneity-free image and shows that $\bar{u}$ can be obtained by dividing $\hat{u}(\mathbf {x})M_N$ by multi-scale intensity information $M_k(\mathbf {x})$. The filter can be named as dual filter formulation, as the image has been filtered twice and then divide it by its average. For a better understanding of the dual filter formulation, we show experimental outcomes, in particular, for a gray-scale synthetic inhomogeneous image and, subsequently, a color image of the plane with relatively high brightness within the background, as shown in Fig. 1. The dual filter is implemented on these two test images with *k* value 10, 20 and 30, respectively, as shown in Figs. 2 and 3.

From Fig. 2, first row third column, it is clear that the intensity inhomogeneity is almost covered but at the same time the edges are also diffuse and an extra region around edges become darker which may cause an unsatisfactory result in segmentation. When the value of *k* increases from $k = 10$ to $k = 20$ and then $k=30$ we notice that the inhomogeneity is slightly removed for higher values of *k* as clear from Fig. 2a,c, but the edges are not affected and an extra region around the edges is not damaged. Figure 3 demonstrates the results of dual-filter formulation on a real-world color image of a plane, which has high brightness in the background due to sunlight which may cause difficulty in segmentation. On the other hand using the filtered image instead of the original one makes the segmentation task easier and more efficient as filter images are clear from the original one. However, it may be noted that the scale parameter *k* plays a vital role in the dual filter formulation, shown in the last column of Fig. 3. As the value of *k* increases, we notice more content in the resulting image but at the same time, it increases the computational cost. Through experiments, we noticed that the *k* value can vary from 5 to 35 and default $k=30$ is more appropriate.

Alternatively, deceptively regularized kernel-oriented techniques have been used to enable local information into the segmentation fitting term. For instance, Elazab et al.^34^ proposed a deceptively regularized and fuzzy kernel-oriented *C*-means clustering (ARKFCM) system and framework. The suggested system has applications in terms of segmentation capabilities for brain MR images and inhomogeneous datasets with the energy function as illustrated mathematically in Eq. (12):

12 $$\begin{aligned} F_{ARKFCM}= & {} 2\sum _{i=1}^N\sum _{j=1}^cu_{ij}^m(1-K(x_iv_j))\nonumber \\&+2\sum _{i=1}^N\sum _{j=1}^c\varphi _i u_{ij}^m(1-K(\hat{x}_iv_j)), \end{aligned}$$

with $x_i$, $i=1:N$, image gray scale in *k* dimensional space, $v_j$, $j=1:c$, cluster center, $u_{i,j}$, the membership value for every pixel *i* and $j{\text{-th}}$ cluster, and *K* the Gaussian radial basis function. Note that in this framework three different algorithms have been suggested that consists of: (i) the local average gray-scaling being substituted by the gray-scale of the mean filter (ARKFCM$_a$), (ii) median filter (ARKFCM$_m$), and (iii) devised weighted images (ARKFCM$_w$), correspondingly. In fact, all these algorithms utilize the heterogeneity of the gray-scales in the pixel locality and, subsequently, put to work this assessment criterion for local contextual information. This should be noted that this is achieved by replacing the standard Euclidean distance with the Gaussian radial basis kernel functions. The ARKFCM framework is independent of parameters which is one of the main advantage of this method and also has promising results for images in presence of noise. Although, one can observe limitation of this technique with images having intensity inhomogeneity, as will be later shown in Fig. 10, usually occurring in MR images.

Recently, Cai et al.^14^ suggested a variational framework for image segmentation while taking advantage of the image restoration techniques. In this work a link among the image segmentation and the image restoration approaches has been shown, whereas Cai et al.^35^ proves arguments on the fact that the solution of CV model^11^ can be achieved through thresholding the minimizer of the ROF model^1^. Finally, the energy functional of the Cai et al. model^14^ is based on two different data fitting terms, i.e., (i) one for image restoration, and (ii) the other one for image segmentation. The relationship is illustrated mathematically as given in the following Eq. (13):

13 $$\begin{aligned} F^{Cai}(u,c_i,v_i)= & {} \mu \int _{\Omega }(u_0-\mathcal {O}u)^2dxdy\nonumber \\&+\lambda \sum _{i=1}^K\int _{\Omega }(u-c_i)^2v_idxdy\nonumber \\&+\sum _{i=1}^K\int _{\Omega }|\nabla v_i|dxdy, \end{aligned}$$

where $\sum _{i=1}^K v_i(\mathbf{{x}})=1,v_i(\mathbf{{x}})\in \{0, 1\}$ is a fuzzy membership function, and $\mathcal {O}$ is a blurring operator i.e., if blur is observed in the image and the aforementioned is a recognition operator for a noisy ascertained image, as well. The blurring operator $\mathcal {O}$ can be computed through using various image de-blurring methods and techniques as suggested in Refs.^36–38^. The Cai et al. model^14^ can efficiently segment images that are damaged and corrupted with the high noise, blur affect, and/or missing pixels; however, its limitation can still be observed in intensity inhomogeneous images, which represents the main problems in the Cai et al. model^14^. This drawback of the Cai et al. method^14^ is due to the fact that the suggested approach and method uses only global information of images and ignores the local one. This issue can be solved through implementing certain machine learning approaches into the image segmentation methods. In next section, we discuss how machine learning based methods can be integrated into existing approaches and use them in the field of image segmentation.

## Machine learning for image segmentation

Deep learning-based method is a newly emerged techniques for image segmentation purposes which has so many types and among them the famous one are: (i) convolutional models with graphical models; (ii) fully convolutional networks; (iii) encoder–decoder based models; and (iv) multi-scale and pyramid network based models^39^. Out of these types, the convolutional neural networks (CNNs) have gained much popularity and extraordinary success in this task of image segmentation. All of these techniques rely on the idea of machine learning approaches and has shown so many promising and excellent results. There are also some method which utilized both active contour and CNN idea to overcome the task of image segmentation, like deep active contour network (DACN) method introduce by Zhang et al.^40^. However, the problem with the CNN approach is that it performs quite badly at identifying precise object boundaries. The major cause is in fact the information loss in the successive down sampling layers^41,42^. On the other hand, the active contour models generate relatively more accurate and useful localization of boundaries by fitting an arch for the object shape in the image using a series of approaches, for instance, (i) the edge based, and (ii) the region based techniques.

In addition to the intensity-based approach, we will discuss the deep learning-based method for tackling low-level computer vision problems, such as de-noising and artifact removal^43^. In Ref.^44^, the authors demonstrate that CNN-based de-noising algorithms, in fact, try to ascertain a mapping function through the maximization of a loss function over a training dataset of the degraded-clean picture pairings. The temporal complexity of the acquisition procedure is quite eminent, despite the fact that this approach is successful and has a short running period. The use of a hierarchical network has improved the learning of high-level features, and thanks to the development of the CNN-based de-noising algorithms. For image segmentation challenges, the Mask RCNN is a deep learning model which is largely used in the existing literature. In fact, this can use the bounding box, classes, and binary image mask to separate various pictures in an image or video. Furthermore, the Faster RCNN was used to create Mask RCNN. For each candidate, F-RCNN produces two outputs: (i) a class label; and (ii) a bounding box. In this paper, we undertake the key and fundamental issue of the intensity inhomogeneity and back light struck images which are still a challenging task for CNN methods as they rely on image average pixels’ values while intensity inhomogeneity is a local property of an image rather than global/average one. Therefore, we rely on active contour along with fuzzy membership function in this article.

Similarly, the long and short term model (LSTM) are also widely used for image segmentation^43^. However, traditional LSTM models are not good because they cannot capture the spatial information of images. Similarly, fully connected weights may significantly increase models computational costs. Therefore, instead of traditional LSTM models the convolutional LSTM methods have been largely used to perform instance-level segmentation. These models can choose every instance of the object in dissimilar timestamps of the sequential result and output. Therefore, to further improve the model performance, attention models are guaranteed as they are supposed to have higher control over the operation of localising particular instances than the traditional convolutional LSTMs, which might choose distinct instances of objects at different timestamps. In Ref.^45^, a deep learning-based denoising technique is demonstrated that incorporates the CNN model with residual connection and attention mechanism. After the Attention-Residual mechanism has calculated the amount of noise in the image, it may be further removed using a simple additive procedure, resulting in the denoised image. A summary of various deep learning based models, including RNN-based methods, can be found in Refs.^39,43,46^.

## The proposed multi-scale-average-filter-assisted Local region restoration segmentation ($M_{SAF}L_{RR}S$) model

Inspired by the well-known Cai et al. model^14^, we suggest a new and novel extension of it which integrates both the global and the local region information into the segmentation of images suffering from intensity inhomogeneity and noise. The proposed work profits from both restoration, as well as, image filtering for segmentation purposes using fuzzy membership function. In difference with Cai model^14^, which utilizes the restored image obtained from ROF formulation, we use multi-scale-average-filter to enable to deal with inhomogeneity toward a accurate image segmentation in the level set formulation. In our proposed method we utilized two types of images namely, $\hat{u}$ and $\bar{u}$ both these images are actually approximation to the given noisy and intensity inhomogeneity image $u_0$. Here $\hat{u}$ represent image free from noise and $\bar{u}$ represents intensity inhomogeneity free image obtained through dual filter formulation. In difference with the Cai et al.^14^ model, which utilizes the restored image obtained from ROF formulation, we use multi-scale-average-filter to enable to deal with inhomogeneity toward accurate image segmentation. By doing so our model is not only capable to tackle noise but also tackle images having diffuse edges, light in background and sever intensity inhomogeneity. In contrast, Cai model is not able to tackle such kind of images as shown and discuss in details in experimental section. The energy functional of the $M_{SAF}L_{RR}S$ model is illustrated in Eq. (14):

14 $$\begin{aligned} F(\hat{u},v_i,k_i,c_i)= & {} \mu \int _{\Omega }(u_0(\mathbf {x}) -\mathcal {O}\hat{u}(\mathbf {x}))^2d\mathbf {x}\nonumber \\&+\lambda _1\sum _{i=1}^K\int _{\Omega }(\hat{u}(\mathbf {x})-k_i)^2v_i(\mathbf {x})d\mathbf {x}\nonumber \\&+\lambda _2\sum _{i=1}^K\int _{\Omega }(\bar{u}(\mathbf {x})-c_i)^2v_i(\mathbf {x})d\mathbf {x}\nonumber \\&+ \sum _{i=1}^K\int _{\Omega }|\nabla v_i(\mathbf {x})|d\mathbf {x}, \end{aligned}$$

where $\sum _{i=1}^K v_i(\mathbf{{x}})=1,v_i(\mathbf{{x}})\in \{0, 1\}$, $\forall x\in \Omega ,$ $\hat{u}\in L^2(\Omega )$ and $\mathcal {O}$ is a linear operator. In this paper, we consider $\mathcal {O}$ as a Gaussian kernel as we have been dealing with Gaussian noise. The first term in Eq. (14) is a image restoration data fitting term. This term removes noise and also controls the closeness of the function $\hat{u}$ to the given image $u_0$. The second and the third terms consist on two fitting terms which utilize both the recover image $\hat{u}$ and the dual-filter image $\bar{u}$ (obtain from Eq. (10)). The third term aims to segment the given image into *K* different intensity levels whereas the second term supports the third term with a fitting to inhomogeneity free clean image. The last term is a TV smoothing term.

This should be noted that the prolongation and revision of the suggested system and model to vector-valued images format is straight forward. Let $\mathbf {u_0}=(u_{01},\cdots ,u_{0p})$, $\hat{\mathbf {u}}=(\hat{u_{1}},\ldots ,\hat{u_{p}})$, $\bar{\mathbf {u}}=(\bar{u_{1}},\ldots ,\bar{u_{p}})$, $\mathbf {k_i}=(k_{i,1},\ldots ,k_{i,p})$ and $\mathbf {c_i}=(c_{i,1},\ldots ,c_{i,p})$, then model (14) can be extended for segmenting vector-valued images as:

15 $$\begin{aligned} F(\hat{\mathbf {u}},v_i,\mathbf {k}_i,\mathbf {c}_i)= & {} \mu \sum _{j=1}^p\int _{\Omega }(u_{0j}-\mathcal {O}_j\hat{u}_j)^2d\mathbf {x}\nonumber \\&+\lambda _1\sum _{i=1}^K\sum _{j=1}^p\int _{\Omega }(\hat{u}_j-k_{i,j})^2v_id\mathbf {x}\nonumber \\&+\lambda _2\sum _{i=1}^K\sum _{j=1}^p\int _{\Omega }(\bar{u}_j-c_{i,j})^2v_id\mathbf {x}\nonumber \\&+\sum _{i=1}^K\int _{\Omega }|\nabla v_i|d\mathbf {x}. \end{aligned}$$

Before minimization of the functional (14) we first relax $v_i$ as; $\sum _{i=1}^K v_i(\mathbf{{x}})=1,v_i(\mathbf{{x}}) \ge 0,$ for all $\mathbf {x} \in$ $\Omega$. Keeping $k_i$, $v_i$, and $c_i$ as constant and deriving with respect to $\hat{u}$ in Eq. (14) we have:

16 $$\begin{aligned} \hat{u}=(\mu \mathcal {O}^T\mathcal {O}+\lambda _1)^{-1}(\mu \mathcal {O}^T u_0+\lambda _1\sum _{i=1}^K k_i v_i). \end{aligned}$$

Similarly, keeping $v_i$, $c_i$ and $\hat{u}$ as constant, and minimize (14) with respect to $k_i$ one have:

17 $$\begin{aligned} k_i=\frac{\int _{\Omega }\hat{u} v_id\mathbf {x}}{\int _{\Omega }v_i d\mathbf {x}}. \end{aligned}$$

Same procedure can be adopted to find the values for $c_i$ by minimizing (14) with respect to $c_i$, for fix $v_i$, $k_i$ and $\hat{u}$:

18 $$\begin{aligned} c_i=\frac{\int _{\Omega }\bar{u} v_id\mathbf {x}}{\int _{\Omega }v_i d\mathbf {x}}. \end{aligned}$$

As discussed in Ref.^14^ that it is possible, and we can prove mathematically, as well as, theoretically, that if $\sum _{j=1}^p\int _{\Omega }(u_{0j}-\mathcal {O}_j\hat{u}_j)^2d\mathbf {x}$ is convex and continuous, then for fixed $k_i$ and $v_i$ there exists only one *u* which minimize the energy consumption using Eq. (14). In order to find $v_i$ with fixed $\hat{u}$ many methods can be adopted, such as Alternating Direction Method of Multipliers^25,47,48^, or applying the primal-dual algorithm^8,49^, or the max-flow approach^50^. For more inside information about finding and estimating the value of $v_i$, interesting readers are referred to Ref.^14^. The steps of the suggested approach is given as follows in Algorithm (1):
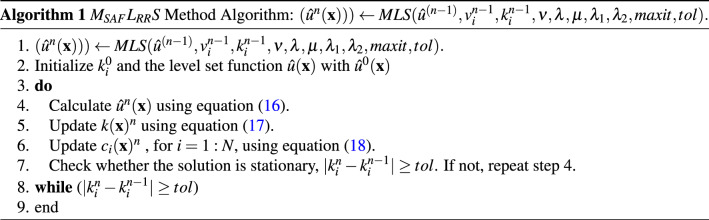


## Experimental results

In this section, we demonstrate and perform numerous numerical experiments to measure and assess the performance of the suggested model. We first show the segmentation accuracy of the suggested model across different synthetic and real dataset images suffering from intensity inhomogeneity in the existence of the noise. We show comparison results of $M_{SAF}L_{RR}S$ model with state-of-art models, for instance, LBF, ARKFCM and Cai models, and show outperforming of the proposed model for real-world images which show complexity due to intensity inhomogeneity, noise and brightness in background. Through numerical experiments, we validate and confirm that the suggested approach is relatively faster and much precise in segmenting images having inhomogeneity and noise. Note that, all the simulations were taken out in the Matlab (R2009b) software on a system with Intel i3 2.2 GHz CPU, 3G RAM, and running the Windows 8 operating system. In all the experiments the values of $\mu$ and $\lambda _1$ were set constant of value 1 except in experiment (Fig. 10), in which value of $\mu$ is taken 50.

The *k*-scale parameter has been set according to the image noise level and intensity inhomogeneity. The maximum number of iterations in each experiment for $M_{SAF}L_{RR}S$ model has been set 100 and the size of images in the range $250 \times 250$ for all the experiments except experiment 9 where the size of image is $150 \times 150$ due to the fact that LBF model produces extra computational cost. The datasets and images used during the experimental study are publicly available in the kaggle repository, and can be accessed online at (https://www.kaggle.com/datasets/mnavaidd/image-segmentation-dataset). This should be noted that we measure the correctness of the suggested model through the factor of Jaccard similarity coefficient^51^ and the Sørensen–Dice similarity index, as described in “Sørensen–Dice similarity” section. In other word, this means that we can assess and measure the similarities among the ground truth *X* and the obtained image *Y* using the Jaccard index. This index is numerically formulated by the following Eq. (19):

19 $$\begin{aligned} J(Y,X)=\frac{|Y \cap X|}{|Y\cup X|}, \end{aligned}$$

where *J* denotes the Jaccard index. The Sørensen–Dice similarity index and its method of computation is described in “Sørensen–Dice similarity” section.

### Accuracy and validation of the proposed model

**Figure 4:**
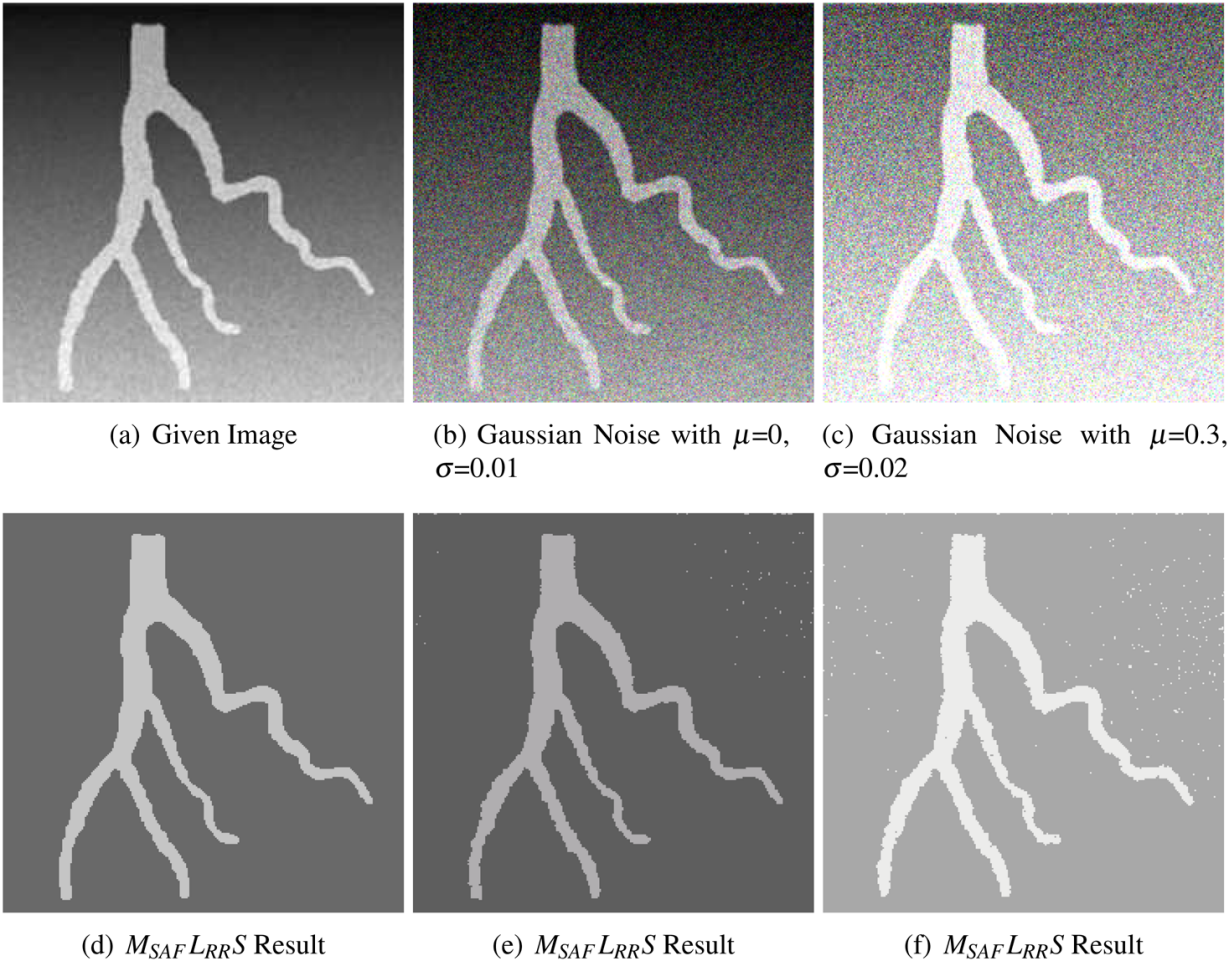
This figure demonstrates the performance of the suggested approach $M_{SAF}L_{RR}S$ on a slight intensity inhomogeneous image in which Gaussian noise is added and increased gradually from left towards right and top towards bottom. The first row represents all those given images that suffered from intensity inhomogeneity and noise while the second row represents the corresponding segmented outcomes of the proposed model. The images were taken from the kaggle repository^33^ and the parameters which were used are $\lambda _2=20$ and $k=32$ (scale parameter).

In order to validate and quantify the performance of the suggested model, we start with a synthetic blood vessel image that is affected from a slight intensity inhomogeneity and has a presence of noise, as shown in Fig. 4a. The Gaussian noise to the blood vessel image varying as: case (1) zero mean and variance 0.01, and case (2) mean 0.3 and variance 0.02, as given in Fig. 4b,c, respectively. From Fig. 4c–e, we can clearly understand that the suggested approach can perfectly segment those given images. Furthermore, this also is clear and evident from various Fig. 4e,f that our anticipated approach has the potentiality and quality to successfully segment the noisy images, very well, as compared to other state-of-the-art models.

**Figure 5:**
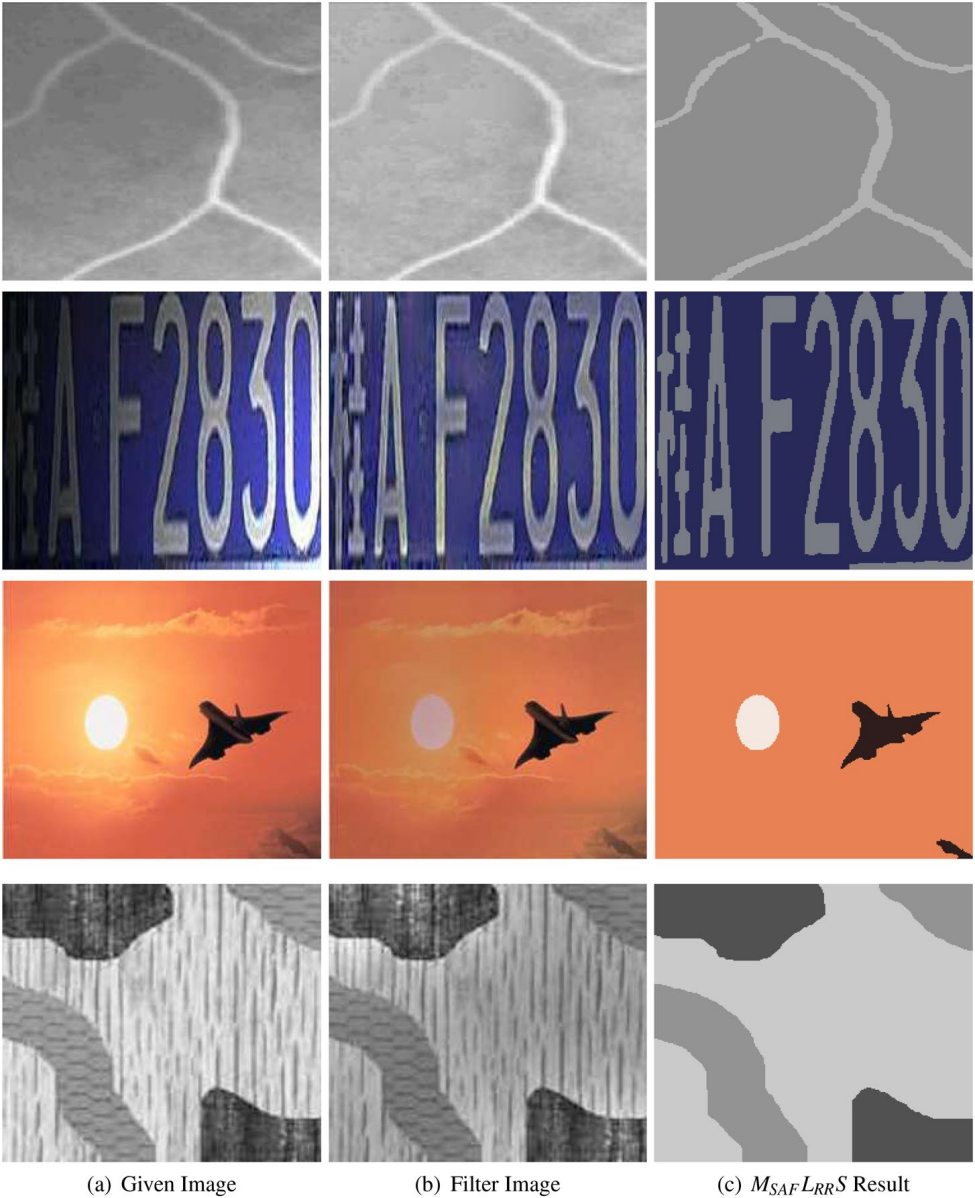
The above figure assesses the performance and effectiveness of our suggested technique on four different images^6,14^. The first column (**a**) shows observed images, the second column, (**b**) shows filter images, while the last column, (**c**) shows the corresponding segmented results. The parameters used for the first row are $\lambda _2=10$ and $k=15$, for the second row $\lambda _2=30$ and $k=10$, for the third row $\lambda _2=100$ and $k=32$, and for the last row $\lambda _2=1$, $k=30$. These inhomogeneous images are taken from Goldstein et al.^6^, Cai et al.^14^ and are publicly available at the kaggle website^33^.

Figure 5 represent experiments of the suggested technique on four different images having intensity inhomogeneity, brightness in background and texture. For instance, the most first column of Fig. 5 displays the given images, second column shows filter images obtained from dual filter formulation while the last column represents the segmented outcomes that were attained with the use of the suggested method. The images taken into account in the first and the second row of this figure are blood vessel and car number plate images which suffer from intensity inhomogeneity and brightness, the third row displays the image of a jet having average intensity background and low and high intensity objects in the foreground, whereas the last row shows a texture image having three different types of texture regions. In fact, this can be easily understood from the very last column of the Fig. 5 that the proposed approach $M_{SAF}L_{RR}S$ has accurately segmented those images.

**Figure 6:**
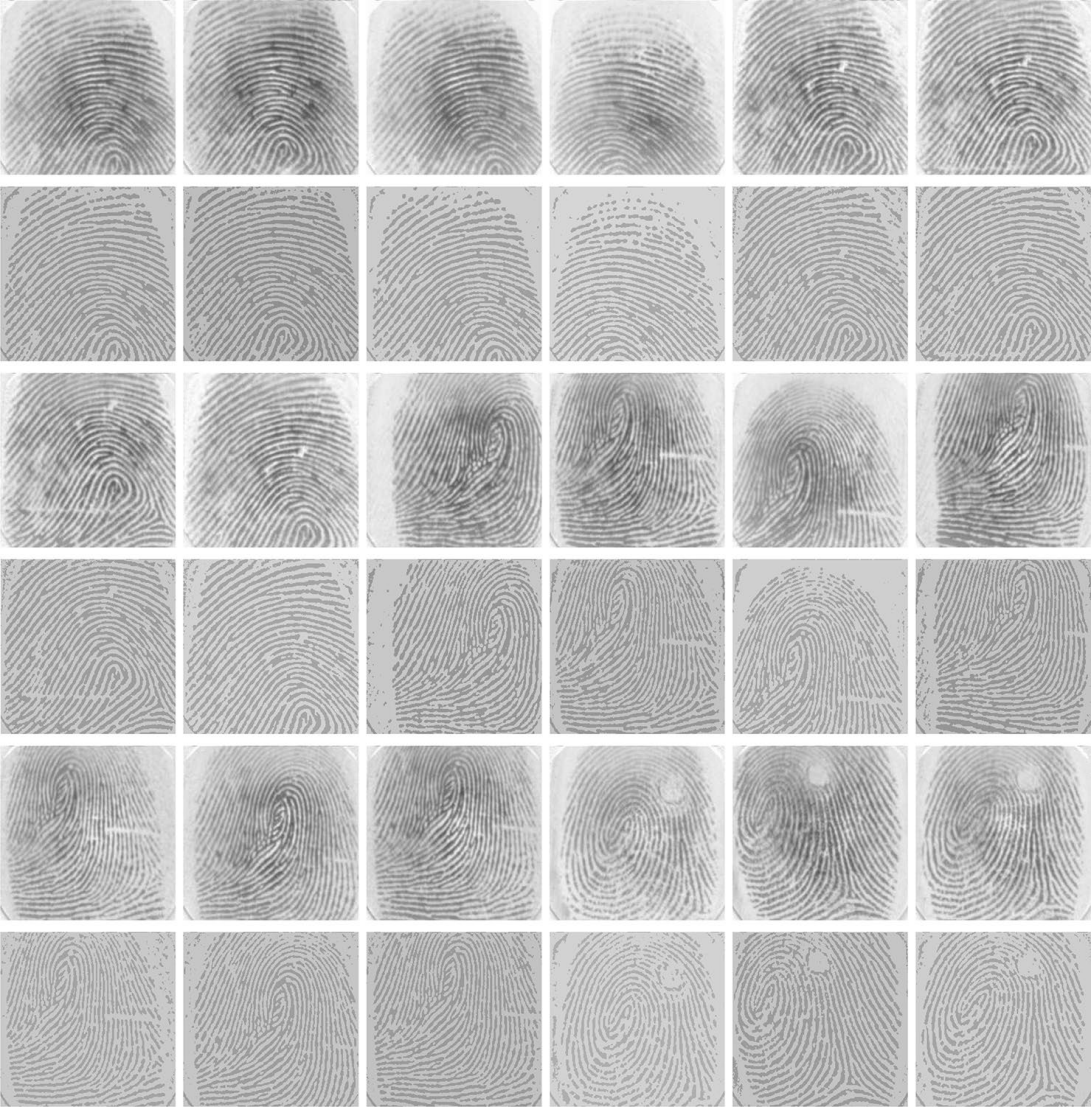
This figure demonstrates the results of our proposed model on a data set of 18 finger prints images. The images were taken from Ref.^33^. The first, third, and fifth rows characterizes the original images while the second, forth and sixth rows characterizes the proportionate segmented results, respectively. In all these experiments the values of various parameters were kept constant which are $\lambda _2=300$ and $k=5$ (scale parameter).

In order to evaluate and further assess the restoration and segmentation performance of the suggested approach, we have employed a public fingerprint image dataset for the purpose. The data set is downloaded from Image Processing Place, Bologna dataset (http://www.imageprocessingplace.com/root_files_V3/image_databases.htm), which consists of eighty images of finger prints with unilluminated foreground and diffuse edges. Due to space limitation, we show in Fig. 6 the results of 18 fingerprint images out of 80. In all the experiments of this dataset, the parameters are kept fixed: $\mu =10$, $\lambda _1=5$, $\lambda _2=300$ and $k=5$(scale parameter) and the maximum number of iterations 100. In the first, third and fifth rows of Fig. 6 we represent the original images whereas the second, fourth and sixth rows represent the corresponding segmented outcomes and findings of the $M_{SAF}L_{RR}S$ model, respectively.

### Comparison of the proposed model with state-of-the-art models

**Figure 7:**
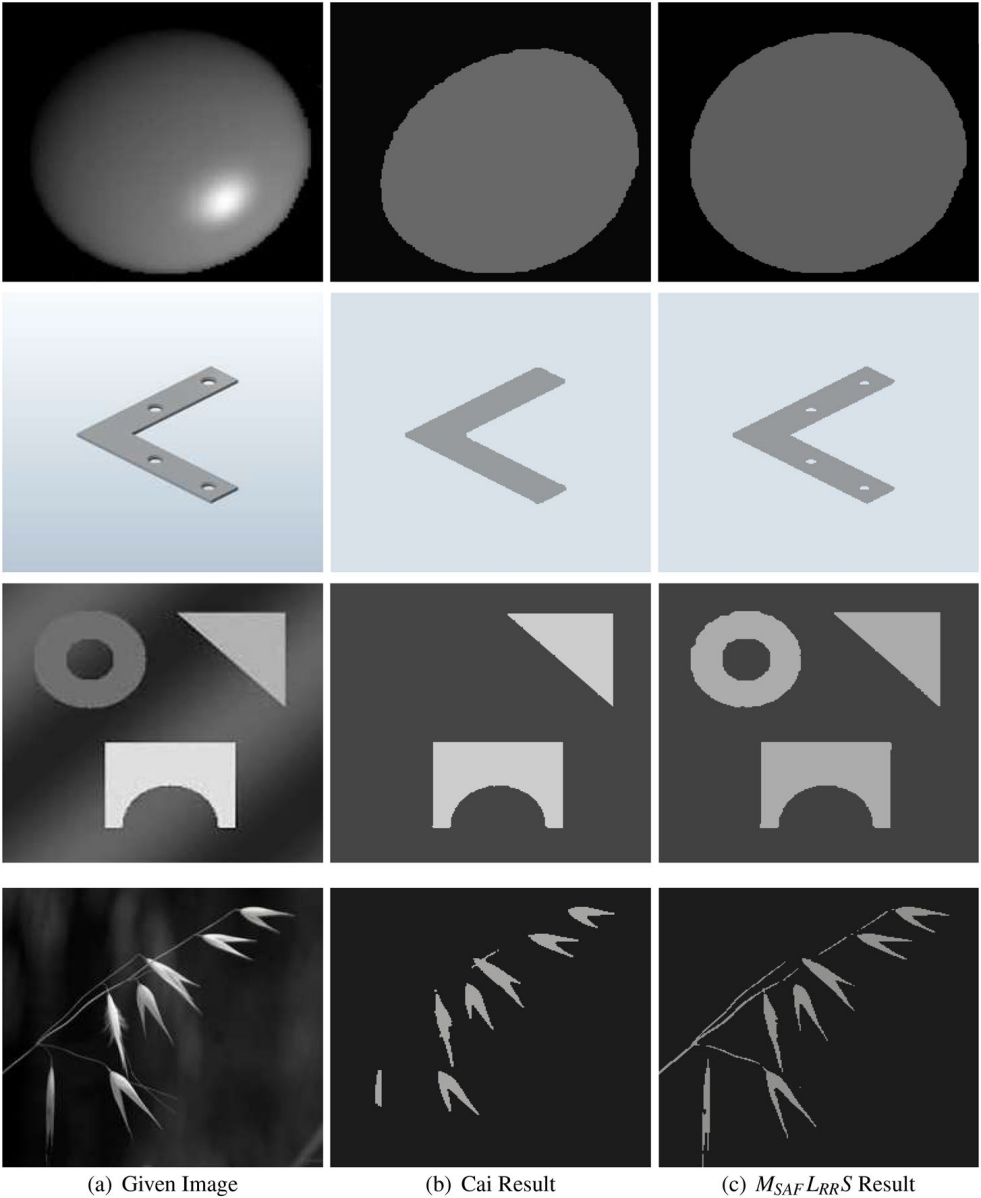
A comparison of the Cai et al.^14^ and the suggested approach on four dissimilar images that are suffered with slender and grievous intensity inhomogeneity^33^. The first column represents given images, however the second and third columns display the segmented results of the Cai et al.^14^ and proposed models, respectively. Moreover, the parameters used for $M_{SAF}L_{RR}S$ model are: (c) $\lambda _2=10$, $k=30$, (f) $\lambda _2=100$, $k=30$, (i) $\lambda _2=50$, $k=35$ and for (l) $\lambda _2=200$, $k=5$.

**Figure 8:**
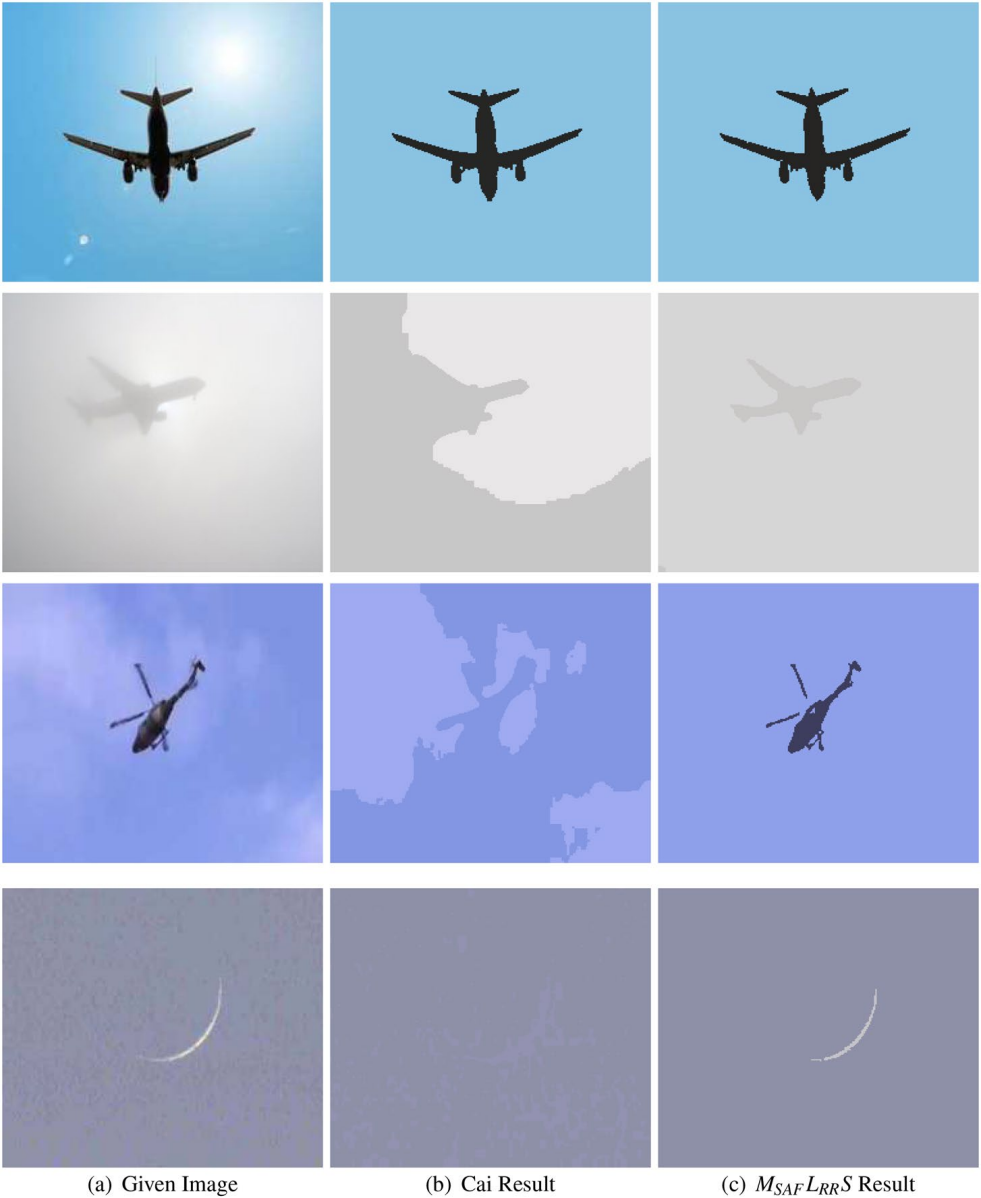
This figure illustrates the results of the Cai et al.^14^ and the suggested approach on four dissimilar color images having brightness, diffuse edges and inhomogeneity^6^. The first column represents given images, while the second and third columns display the segmented results of the Cai et al.^14^ and the proposed models, respectively^14^. Note that the parameters used for $M_{SAF}L_{RR}S$ model are: first row image $\lambda _2=50$, $k=32$, second row image $\lambda _2=500$, $k=10$, third row $\lambda _2=100$, $k=30$ and the last row image $\lambda _2=500$, $k=30$. These images were taken from the kaggle datasets^33^ and are available publicly.

We will compare the suggested approach with other state-of-art models including, LBF^16^, Cai^14^, and ARKFCM^34^, and illustrates the experimental results. For a better apprehension of the advantage of the anticipated technique, we start its comparison with the stimulus model of the Cai et al.^14^. Figures 7 and 8 show some synthetic and real image data (first column) processed with Cai et al. method^14^ and the proposed method. The results obtained from the Cai et al. method^14^ are given away in the second column whereas the outcomes obtained with our model in the last column. We can intelligibly see that the suggested approach, similar to the Cai et al. method^14^, not only can easily deal with such images but furthermore, the method shows a perfect accuracy of the segmented images by including all the object’s details. The images considered in Figs. 7 and 8 suffer from intensity inhomogeneity and having unilluminated objects boundaries. The first row of Fig. 7 is a synthetic image with a bright portion suffered from intensity inhomogeneity. We can clearly understand that the suggested and anticipated approach has successfully segmented the object of this image whereas we see a partially successful result of the Cai et al. model^14^. The second row of this figure shows the results of synthetic images suffered from slight and severe intensity inhomogeneity, respectively. Form the second row second and third column of Fig. 7 it is clearly seen that both the Cai et al. model^14^ and the proposed model perform very well in segmenting the overall object of the iron bar but on the other hand, it may be noted that the Cai et al. model^14^ fails to segment the interior circle in the object while $M_{SAF}L_{RR}S$ successfully segments them. The third row of Fig. 7 consists of a multilevel intensity image with grievous intensity inhomogeneity. Nevertheless, in order to segment such an image we use the supposition that the given image has two different levels of intensity of the objects to be segmented. To proceed with the segmentation of such images a multi-phase segmentation can be used, specifically two phase image i.e., the value of *K* (number of phases) is two for both the $M_{SAF}L_{RR}S$ and the Cai et al. model^14^. We clearly see from the third row in Fig. 7 that the Cai et al. model^14^ (second column) extracts only two objects while $M_{SAF}L_{RR}S$ (third column ) extracts all the three objects. The last image of Fig. 7 is a plant image having diffuse branches. The last row (second and third column)in Fig. 7 shows that the Cai et al. model^14^ segments only the leaves and is unable to segment its branches while on the other hand $M_{SAF}L_{RR}S$ method can capture both of them.

Figure 8 demonstrates the results of the Cai at al.^14^ and $M_{SAF}L_{RR}S$ model on four real-world test images, in which the first image to be tested is a plane image with sun light, the second image is a plane image with diffuse edges, while the third and the last images are helicopter and crescent moon images with unclear edges. In the first experiment of Fig. 8 (shown in the first row) both Cai^14^ and $M_{SAF}L_{RR}S$ models perform very well. In the second row of this figure, we can see that Cai model^14^ fails to segment the image of the plane with diffuse edges whereas the proposed model has good segmentation outcomes (as given away in the second row last column of Fig. 8). In addition, the last column of the third and the forth row of Fig. 8 clearly demonstrate the robustness of our proposed model $M_{SAF}L_{RR}S$ over the Cai et al. model^14^, i.e., a total fail of Cai model^14^ for such images. In fact, the proposed model $M_{SAF}L_{RR}S$ segments very efficiently the edges as seen in Fig. 8.

**Figure 9:**
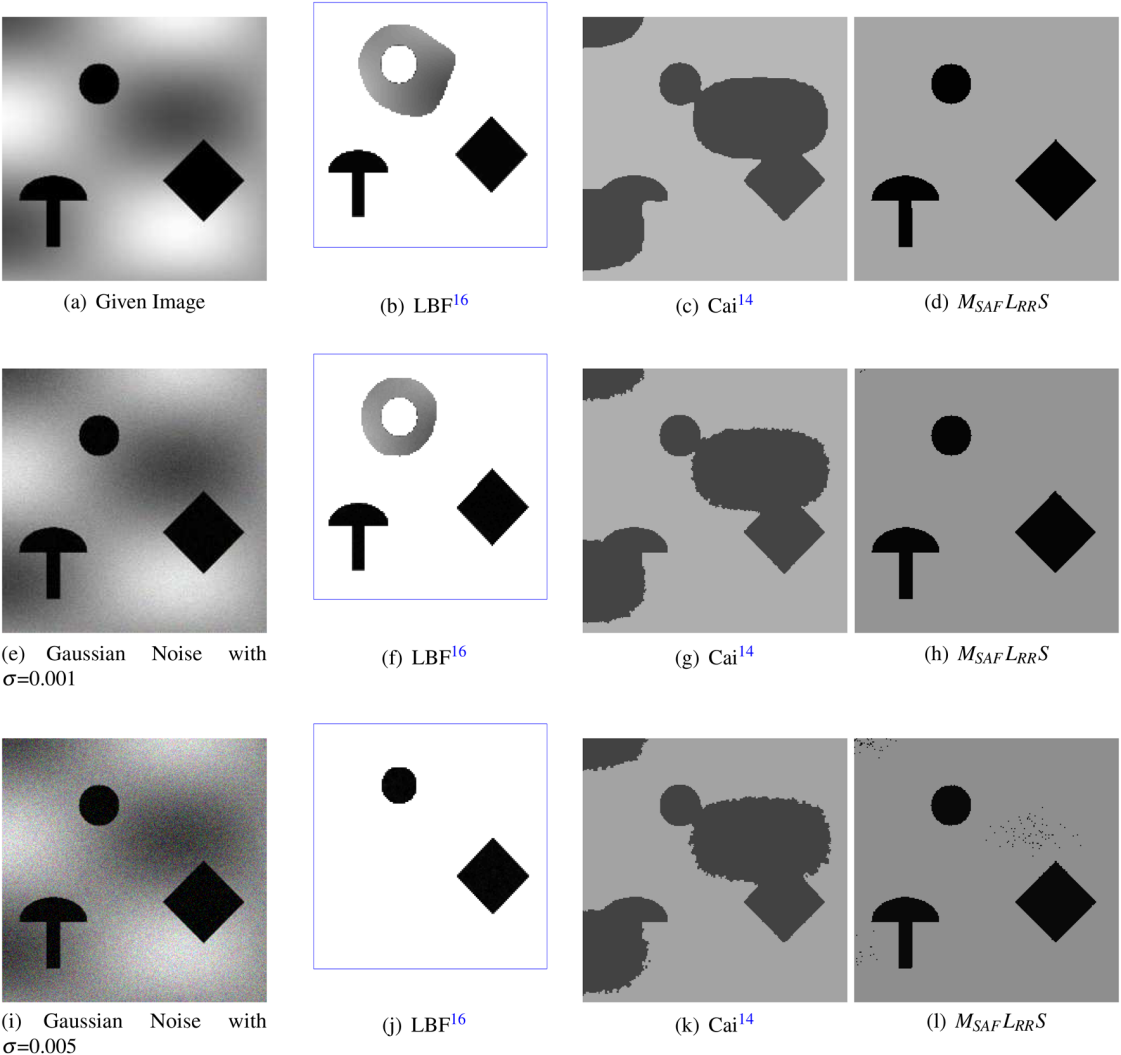
Segmenting a synthetic image, using the LBF approach, Cai et al.^14^ method, and the suggested $M_{SAF}L_{RR}S$ approach. The first column represents the original image and its noisy version, while the second column characterizes the result of the LBF method. Furthermore, the third and forth columns represent the segmented outcomes of the Cai et al.^14^ and proposed model, respectively. Furthermore, the parameters used for $M_{SAF}L_{RR}S$ model are (d) $\lambda _2=100$, $k=10$ and for (h) and (l) are $\lambda _2=200$, $k=20$. The images were taken from Ref.^33^ and are publicly available.

In Fig. 9, the first, second, third and fourth column represent the original image, the obtained results with LBF model^16^, Cai et al. model^14^, and outcomes of the anticipated technique, respectively. The first row of this figure comparisons the findings for a synthetic image with slight intensity inhomogeneity. In the second and third row Gaussian noise is added with mean zero and variance 0.001 (second row) and 0.005 third row, respectively. From the last column in Fig. 9, we can notice that $M_{SAF}L_{RR}S$ performs very well in all the three test images as compared to the LBF^16^and the Cai et al. models^14^. The imperfect results of the LBF^16^ and the Cai et al. model^14^ can be observed clearly in second and third column, correspondingly.

**Figure 10:**
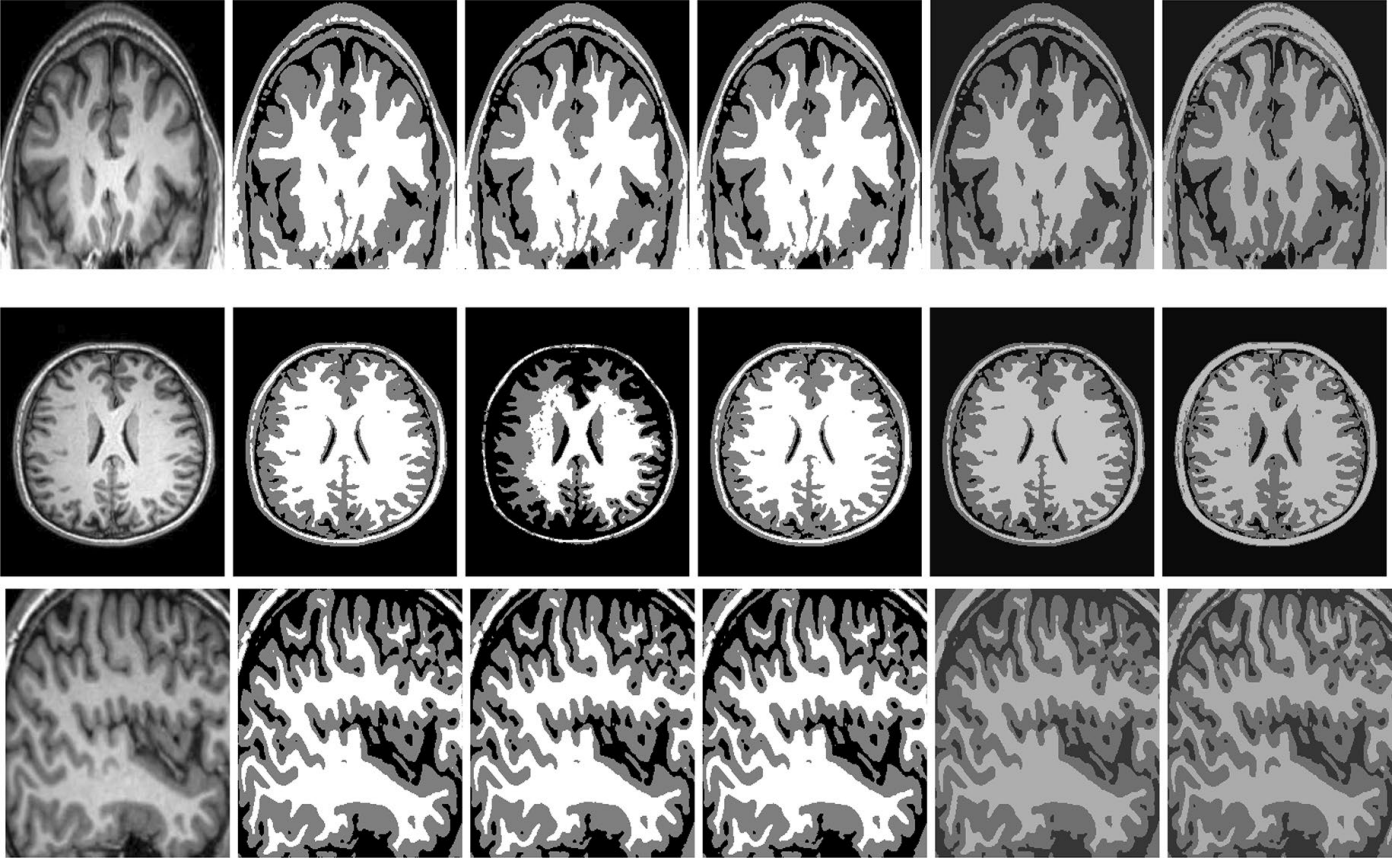
Segmentation results on three dissimilar MR images of the brain. Note that, these images were taken from the kaggle repository^33^. The first column out of the six columns represents original images, while second, third and fourth columns represent segmentation outcomes of ARKFCM$_a$, ARKFCM$_m$ and ARKFCM$_w$, respectively. Similarly, the fifth and the last column demonstrates segmented results of the Cai et al.^14^ and $M_{SAF}L_{RR}S$ models, respectively. In all these three experiments (three rows), the parameters used for our proposed model are $\lambda _2=30$ and $k=30$.

Figure 10 demonstrates the comparison among ARKFCM method^34^ with different waited filters (ARKFCM$_a$, ARKFCM$_m$ and ARKFCM$_w$), Cai et al. method^14^ and the anticipated $M_{SAF}L_{RR}S$ model. The first column of this figure displays the given images, the second, third and the fourth columns display all segmented outcomes with ARKFCM$_a$, ARKFCM$_m$ and ARKFCM$_w$, respectively, whereas the fifth and last columns show segmented results of Cai and $M_{SAF}L_{RR}S$ methods, respectively. Figure 10 shows comparison of those methods on three brain MR images which suffer from intensity inhomogeneity. As ARKFCM framework consists of three methods i.e. average, mean and weighted, therefore we tested each image of Fig. 10 we compare our model with all of them. In fact, this can be well understood from all outcomes and these figures that all the three methods of ARKFCM framework are unable to segment the given brain MR images by missing significant details in it. Similarly, in the fifth column of this figure we can see that Cai model^14^ also is unable to segment these images properly. In contrast, the segmented results of $M_{SAF}L_{RR}S$ model (last column) capture all the significant details present in these MR images of brain.

The Jaccard similarity measure, and the CPU time (which is measured in seconds) of the proposed and other state-of-the-art models are given away in Table 1. The statistics deliberate that the anticipated model is better than other state-of-the-art model, including the well-known Chan-Vese^11^ and Cai et al.^14^ models, in terms of higher Jaccard similarity (JS) ratio and lower CPU or computational time. This should be noted that variations in the JS values and CPU times are, in fact, due to the heterogeneity of the images and their different characteristics.

**Table 1:** The Jaccard similarity measure, and CPU time (seconds) of Cai et al.^14^, ARKFCM^34^, Chan-Vese^11^, LBF^16^, and of the anticipated approach on 10 different images from the Berkeley’s dataset.

Image	Proposed model		Chan-Vese^11^		Cai et al.^14^		LBF^16^		ARKFCM^34^	
	JS	Time	JS	Time	JS	Time	JS	Time	JS	Time
1	0.999	0.068	0.912	0.095	0.755	0.195	0.206	0.185	0.725	0.595
2	0.872	0.084	0.862	0.195	0.747	0.105	0.780	0.095	0.777	0.175
3	0.921	0.085	0.890	0.123	0.476	0.173	0.303	0.035	0.890	0.203
4	0.789	0.084	0.792	0.153	0.722	0.143	0.403	0.097	0.922	0.253
5	0.720	0.071	0.679	0.157	0.506	0.174	0.547	0.096	0.588	0.096
6	0.919	0.066	0.588	0.096	0.547	0.175	0.363	0.098	0.527	0.175
7	0.773	0.081	0.542	0.082	0.440	0.141	0.283	0.103	0.689	0.152
8	0.784	0.075	0.789	0.152	0.732	0.152	0.521	0.073	0.547	0.096
9	0.858	0.061	0.825	0.123	0.635	0.153	0.345	0.093	0.863	0.113
10	0.813	0.071	0.732	0.183	0.652	0.153	0.431	0.150	0.521	0.073

### Sørensen–Dice similarity

Besides the Jaccard similarity coefficient other quantitative can also be implemented to assess and quantify the performance of the proposed image segmentation approach. The Sørensen–Dice similarity values are normalized and given with in the limit of [0, 1]. The greater value for the Dice shows relatively superior segmentation outcomes and vice versa. Very similar to the Jaccard similarity coefficient, the Sørensen–Dice similarity index value is computed using the following Eq. (20):

20 $$\begin{aligned} D(Y,X)=\frac{2|Y \cap X|}{|Y|+|X|}, \end{aligned}$$

where *X* represents the ground truth, *Y* denotes the obtained image and *D* shows the Sørensen–Dice similarity between *Y* and *X*. Table 2 shows the Sørensen–Dice coefficients values for comparison purposes of the anticipated approach with other competitory techniques, i.e., Cai et al.^14^, LBF^16^, Chan-Vese^11^, and ARKFCM^34^. This should be noted that these results were attained using numerical simulations on 10 various images that were in fact appropriate for interactive segmentation using a particular predefined ground truth value. Note that the value is assumed to be comprising means of the ground truth. This can be determined that the Cai et al. model generated comparatively superior outcomes as equated to the LBF and ARKFCM models. However, for a richly noisy or depleted intensity image the anticipated approach loses the statistics. From the outcomes, as shown in Table 2, this is clear that the suggested approach performs superior to the other competing techniques. The Chan-Vese^11^ performs relatively better than the Cai et al.^14^ model but the Sørensen–Dice similarity value of the suggested approach is still higher than the Chan-Vese model^11^. In fact, the higher values of the deviations show that the model perform completely different over various images.

**Table 2:** The Sørensen–Dice similarity for the Cai et al.^14^, LBF^16^, Chan-Vese^11^, ARKFCM^34^ and of our suggested approach on 10 dissimilar images.

State-of-the-art models				Suggested model
Cai et al.^14^	LBF^16^	ARKFCM^34^	Chan-Vese^11^	
0.959 ± 0.079	0.936 ± 0.067	0.814 ±0.069	0.961 ± 0.072	0.986 ± 0.084

## Conclusions and future work

In this paper, we developed a new hybrid variational approach for image segmentation, restoration, and filtering. The planned approach is especially tailored for those images that suffer with the intensity inhomogeneity, noise or brightness in the background. For this purpose we took advantage of utilizing dual filter formulation and fuzzy membership function. We tested our proposed model on a variety of images including real life images of plane and public data set of fingerprints images and observed that the proposed model can tackle weak fingerprint images very well. We also proved that our model is more accurate and fast as compared to available models used for the same task. We observed in empirical evaluation and through the obtained outcomes that the proposed model $M_{SAF}L_{RR}S$ segments the edges of various images very efficiently and accurately. we compared our proposed model with other well known and latest models used for image segmentation and proved that our method perform better than these methods. Our future goal is to modify the proposed model for severe blurry and foggy images as these are difficult to segment and restore with the existing methods.

One of the most fundamental fields in real-world applications and medical imaging is selective image segmentation. In the future, we will work on and deliver a strong selective segmentation approach grounded on the concept of local spatial distance; and simultaneously utilising a dual-level set variational formulation model. A comparable approach should attempt to divide all image items through a single level set function (aka. global) and the chosen item through a dissimilar level set function (aka. local). Additionally, in the future, the use of a mix and amalgamation of marker distance function, and local spatial distance. Nevertheless, we will continue investigating the edge detection, and active contour without edges should also be examined in parallel. In the existence of outliers and noise, especially the Gaussian noise, outliers should be recognised and separated during the pre-processing of denoising. This should be kept in mind that suitable constraints should be put forward and assumed to the segmentation framework in order to ensure and guarantee proper and acceptable picture segmentation. In the future, we will use more appropriate, robust, and appropriate approaches for removing outliers’ and criteria, in particular, integrated with and backed by a well-designed hypothesis in a variational model for precise and acceptable image restoration and segmentation.

## Data Availability

The datasets generated and/or analysed during the current study are publicly available in the kaggle repository, and can be accessed at [https://www.kaggle.com/datasets/mnavaidd/image-segmentation-dataset]. Moreover, various images used within the experimental work are publicly available online. All the codes used for this method will be provided for research purposes if requested by researchers.
